# Factores asociados con la infección por el virus de la hepatitis B en comunidades indígenas de Colombia

**DOI:** 10.7705/biomedica.7243

**Published:** 2024-05-30

**Authors:** Jaime Martínez-Gallego, Diana Castro-Arroyave, Juan Carlos Quintero, Fernando de la Hoz, Melissa Montoya, Isabela Palacio, María Cristina Navas, Carlos Rojas

**Affiliations:** 1 Grupo de Epidemiología, Facultad Nacional de Salud Pública, Universidad de Antioquia, Medellín, Colombia Universidad de Antioquia Universidad de Antioquia Medellín Medellín; 2 Grupo de Estudio en Pedagogía, Infancia y Desarrollo Humano - GEPIDH, Facultad de Educación, Universidad de Antioquia, Medellín, Colombia Universidad de Antioquia Universidad de Antioquia Medellín Medellín; 3 Facultad de Medicina, Universidad Nacional de Colombia, Bogotá, D. C., Colombia Universidad Nacional de Colombia Universidad Nacional de Colombia Bogotá D. C Bogotá; 4 Grupo de Gastrohepatología, Facultad de Medicina, Universidad de Antioquia, Medellín, Colombia Universidad de Antioquia Universidad de Antioquia Medellín Medellín

**Keywords:** hepatitis B, factores de riesgo, pueblos indígenas, salud de poblaciones indígenas, estudios de casos y controles, Hepatitis B, risk factors, indigenous peoples, health of indigenous populations, case-control studies

## Abstract

**Introducción.:**

Colombia alberga dos millones de indígenas, que viven en condiciones de pobreza y tienen deficiencias en salud, por lo cual están expuestos a contraer infecciones virales como la hepatitis B. El departamento del Amazonas presenta una gran prevalencia del virus y barreras para acceder a la vacunación; por esto, parte de la población es propensa a la infección.

**Objetivo.:**

Identificar factores asociados con la infección por el virus de la hepatitis B en indígenas colombianos.

**Materiales y métodos.:**

Se llevó a cabo un estudio de casos y controles en mayores de 18 años de cuatro departamentos del país. Los casos se identificaron mediante el registro nacional de notificación de hepatitis B (2015-2022). Los controles seleccionados de manera concurrente fueron pareados con los casos por edad, sexo, etnia y departamento. En una encuesta se consignaron las características sociodemográficas, los factores asociados con el contacto con sangre y fluidos, las prácticas socioculturales y los antecedentes de vacunación. El proyecto fue aprobado por Comité de Ética de la Universidad de Antioquia.

**Resultados.:**

Participaron 75 casos y 150 controles de 13 grupos étnicos. El departamento del Amazonas aportó el 49 % de los participantes (83 % mujeres) con una mediana de edad de 30 años (RIC = 27-37). Los factores asociados con una mayor probabilidad de contraer la infección fueron el antecedente de algún familiar infectado con el virus de la hepatitis B (OR ajustado = 2,61) (IC_95%_: 1,09-6,27) y número de embarazos en mujeres, (OR ajustado = 1,61) (IC_95%_: 1,02-2,54). La vacunación mostró un efecto protector sin asociación significativa.

**Conclusión.:**

Los aspectos asociados con la convivencia familiar y el número de embarazos contribuyen a una potencial transmisión vertical y horizontal del virus. No se identificaron prácticas culturales asociadas. Se requieren estrategias novedosas y diferenciales para reducir la transmisión del virus de la hepatitis B en poblaciones indígenas.

## Abstract (en)

**Introduction.:**

Colombia is home to 2 million indigenous people who live in conditions of poverty and with health deficiencies, making them vulnerable to contracting hepatitis B (HBV). Amazonas has a high virus prevalence, and there are barriers to accessing vaccination; thus, part of the population is susceptible to infection.

**Objective.:**

To identify factors associated with HBV in Colombian indigenous people.

**Materials and Methods.:**

A case-control study of people over 18 years from four departments of Colombia. Cases were identified through the national hepatitis B notification registry (2015-2022). Controls were selected and matched to cases (2:1) by age, sex, ethnicity, and department. Sociodemographic characteristics, factors associated with contact with body fluids, cultural practices, and vaccination history were identified by means of a survey. The ethics committee of the *Universidad de Antioquia* approved the project.

**Results.:**

Seventy five cases and 150 controls from 13 ethnic groups were surveyed. Amazonas contributed 49% of participants, 83% were women, and the median age of cases was 30 years (IQ range: 27-37). The associated factors were a family history of hepatitis B [adjusted OR: 2.61 (95% CI: 1.09-6.27)] and, in women, the number of pregnancies [adjusted OR: 1.61 (95% CI 1.02- 2.54)]. The vaccination history showed a protective effect, but the association was not significant.

**Conclusion.:**

Aspects associated with family life and unprotected sexual relations seem to be responsible for the potential transmission of the virus. It was not possible to identify associated cultural practices. Innovative and differential strategies are required for indigenous people to achieve a reduction of HBV.

En Colombia, existen aproximadamente dos millones de indígenas pertenecientes a 105 grupos étnicos, cada uno con su propia lengua y cultura ^1^^,^^2^. La mayoría vive en zonas rurales, en condiciones de pobreza, marginación, discriminación y con estados de salud muy deficientes comparados con el del resto de la población, lo que hace que sean más propensos a contraer enfermedades infecciosas ^1^^,^^3^. La hepatitis B, cuyo agente etiológico es el virus de la hepatitis B (HBV), es una de las enfermedades de las que se reportan casos en indígenas desde mediados del siglo XX ^4^.

En el departamento del Amazonas, se ha presentado gran prevalencia e incidencia de esta infección y se han reportado brotes de hepatitis fulminante asociados con sobreinfección o coinfección con el virus de hepatitis delta ^5^^,^^6^.

Para responder a esta amenaza de salud pública, Colombia inició desde 1992 el programa de vacunación contra el virus de la hepatitis B en indígenas recién nacidos y niños menores de cinco años de los departamentos de Amazonas, Cesar y Norte de Santander, estrategia que se extendió por el resto del país ^7^. Los estudios realizados en regiones indígenas mostraron un descenso en la prevalencia de la infección y los portadores del antígeno de superficie, aunque aún existen barreras para el acceso al esquema completo de vacunación y, como consecuencia, parte de la población sigue en riesgo de contraer la infección ^8^^-^^10^.

La transmisión vertical del virus de la hepatitis B es frecuente en el Amazonas, aunque la transmisión horizontal mediante fluidos sanguíneos y secreciones también es clave en esta población ^11^^-^^13^.

El objetivo de este estudio fue identificar los factores sociodemográficos y socioculturales asociados con la infección por el virus de la hepatitis B en indígenas del Amazonas, Guaviare, La Guajira y Antioquia, para generar nuevos conocimientos y desarrollar estrategias de prevención y atención -culturalmente pertinentes- que disminuyan la incidencia del virus de la hepatitis B en las poblaciones indígenas y aporten a la meta de desarrollo sostenible de “eliminar las hepatitis virales para el 2030” ^14^.

## Materiales y métodos

### Áreas de estudio

Se incluyeron indígenas de los departamentos del Amazonas, Guaviare, La Guajira y Antioquia. El departamento del Amazonas y del Guaviare se seleccionaron dada la gran incidencia de hepatitis B según los registros del Instituto Nacional de Salud de Colombia ^9^. Antioquia y La Guajira no presentan una incidencia alta de virus de la hepatitis B, pero son departamentos donde los investigadores tienen estudios previos y, con ello, acceso a comunidades indígenas y apoyo de organizaciones y entidades promotoras de salud indígena en calidad de aliados.

### Tipo de estudio

Se diseñó un estudio pareado de casos y controles, con una selección de controles mediante muestreo por densidad, pareados por departamento, sexo, etnia y edad. Por cada caso detectado se asignaron dos controles. Los casos se definieron como personas indígenas mayores de 18 años, hispanohablantes, con prueba positiva -inmunoensayo o prueba rápida- para antígeno de superficie de hepatitis B (HBsAg), reportada en el Sistema de Vigilancia en Salud Pública (SIVIGILA), ente encargado de la provisión sistemática de la información de los eventos de interés en salud pública en Colombia.

La información de los casos notificados de hepatitis B fue suministrada por las secretarías de salud y las empresas prestadoras de servicios de los departamentos participantes, entre el 2015 y el 2022, y se recolectó de manera periódica hasta el fin del intervalo.

Los pacientes fueron localizados en cada departamento participante y se contactaron con ayuda del personal de salud de cada región. Debido a limitaciones logísticas y al confinamiento por la pandemia de COVID-19, inicialmente se buscaron los casos que residían en la zona urbana de las ciudades principales de cada departamento.

Los controles se seleccionaron en las comunidades; fueron indígenas mayores de 18 años, hispanohablantes y sin infección por el virus de la hepatitis B, dato confirmado y reportado mediante una prueba rápida con resultado negativo. La selección se hizo de manera concurrente una vez detectados los casos. Las personas indígenas que cumplían con los criterios de inclusión (familiares, vecinos, allegados) fueron invitados a participar de manera voluntaria. Todos los participantes del estudio fueron tamizados con una prueba rápida para detección del virus de inmunodeficiencia humana (HIV), para identificar potenciales casos de coinfección.

### Muestra y muestreo

Se calculó un tamaño de muestra de 67 casos y 134 controles, con el que podría detectarse como significativa una razón de probabilidades (*odds ratio*, OR) igual o superior a 2,5, con un nivel de confianza de 95 % y un poder estadístico del 80 %. Se asumió una frecuencia de exposición del 20 % en el grupo control (esta asunción es hipotética y se adscribió a todos los posibles factores por identificar dada la escasez de estudios previos que reportaran este dato). En algunos casos, los controles fueron elegidos en los centros de salud con apoyo del personal idóneo de las instituciones.

### Recolección de información

Se aplicó una encuesta epidemiológica de 75 preguntas organizadas en siete bloques temáticos: información sociodemográfica, aspectos socioculturales, historia de contacto con fluidos corporales, prácticas sexuales, conocimientos de la enfermedad, estado de salud y antecedente de vacunación. El cuestionario fue revisado por expertos antes de su utilización y, con ayuda de asistentes de investigación previamente entrenados, se ejecutó un estudio piloto con personas indígenas, miembros de una organización indígena en Medellín.

### Análisis de información

La información obtenida se almacenó en una base de datos, creada con el *software* Epi Info^TM^, versión 7.2.4, con la que se realizaron los análisis estadísticos descriptivos de las principales características sociodemográficas y socioculturales, y las conductas de riesgo de los participantes.

Se calcularon frecuencias relativas y absolutas para las variables cualitativas, y medidas de tendencia central, posición y dispersión, para las cuantitativas. Se estimaron factores asociados con la infección por el virus de la hepatitis B mediante una regresión logística mixta, donde la variable de agrupación de los casos -sexo, edad, etnia y departamento- se utilizó como efecto aleatorio de los modelos.

Se construyeron modelos bivariados para seleccionar las variables candidatas a conformar el análisis multivariado, considerando aquellas que tuvieran un valor de p menor o igual a 0,25. Además, en el análisis multivariado se incluyeron las variables que tuvieran la posibilidad biológica de relacionarse con la infección por el virus de la hepatitis B. Se realizó un modelo de regresión logística mixta solo con mujeres, para investigar las exposiciones relacionadas con antecedentes reproductivos.

El modelo multivariado final se seleccionó teniendo en cuenta la metodología paso a paso, el concepto de los investigadores y los criterios de información de Akaike. Todos los análisis estadísticos se calcularon con los paquetes estadísticos Stata™, versión 16.1 y SAS™, versión 3.1.0.

### Consideraciones éticas

El estudio y los investigadores se acogieron a las guías internacionales para la investigación con seres humanos, consignadas en la última versión de la Declaración de Helsinki de la Asociación Médica Mundial, y a las nacionales, según las resoluciones 008430 de 1993 y 2.378 de 2008 del Ministerio de Salud y Protección Social, que abordan las técnicas y recomendaciones para la investigación en salud y las buenas prácticas clínicas.

Los casos y los controles participaron de manera voluntaria en el estudio y firmaron un consentimiento informado antes del diligenciamiento de la encuesta, la toma de la muestra y la aplicación de las pruebas rápidas para el HIV y el virus de la hepatitis B. La investigación contó con el aval del Comité de Ética de la Facultad Nacional de Salud Pública de la Universidad de Antioquia, como consta en el acta CI00196-2017.

## Resultados

### Características sociodemográficas de los participantes

Se incluyeron 75 casos y 150 controles, con un muestreo adicional de 8 casos y 16 controles. Se incluyeron 37 casos y 74 controles del departamento del Amazonas, 18 casos y 36 controles de La Guajira, 15 casos y 30 controles del Guaviare, y 5 casos y 10 controles de Antioquia. El 82,7 % de los participantes fueron de sexo femenino, con una mediana de edad de 30 años (RIC = 27-37) para los casos y de 31 años (RIC = 25-40) para los controles. Participaron 13 grupos étnicos: ticuna, el 30,7 %; wayuu, el 34 %; nukak, el 14,7 %, y yagua el 13,3 % (cuadro 1).

**Cuadro 1:** Características sociodemográficas de casos y controles

Variable		Casos (N = 75) n (%)	Controles (N = 150) n (%)
Sexo			
	Femenino	62 (82,7)	124 (82,7)
	Masculino	13 (17,3)	26 (17,3)
Edad (años) (mediana y RIC)		30 (27-37)	31 (25-40)
Departamento			
	Amazonas	37 (49,3)	74 (49,3)
	La Guajira	18 (24,0)	36 (24,0)
	Guaviare	5 (20,0)	30 (20,0)
	Antioquia	5 (6,7)	10 (6,7)
Etnia indígena			
	Ticuna	23 (30,7)	49 (32,7)
	Wayuu	18 (24,0)	36 (24,0)
	Nukak	11 (14,7)	16 (10,7)
	Yaguas	10 (13,3)	14 (9,3)
	Emberá	5 (6,7)	10 (6,7)
	Cocama	2 (2,7)	8 (5,3)
	Tucano	1 (1,3)	3 (2,0)
	Jiw	2 (2,7)	9 (6,0)
	Cubea	0 (0)	2 (1,3)
	Tanimuca	0 (0)	2 (1,3)
	Andoque	2 (2,7)	1 (0,7)
	Yuruti	1 (1,3)	0 (0)
Estado civil			
	Casado/unión libre	58 (77,3)	121 (80,7)
	Soltero	15 (20,0)	24 (16,0)
	Divorciado	1 (1,3)	4 (2,7)
	Viudo	1 (1,3)	0 (0,0)
	No responde	0 (0)	1 (0,7)
Escolaridad			
	Primaria	31 (41,3)	49 (32,7)
	Secundaria	24 (32,0)	58 (38,7)
	Educación superior	11 (14,7)	32 (21,3)

RIC: rango intercuartílico

Estar casado o vivir en unión libre fueron las categorías de estado civil más frecuentes, con el 77,3 % para los casos y el 80,7 % para los controles. En nivel de escolaridad, el 41,3 % de los casos tenía estudios de básica primaria y, de los controles, solo el 32,7 %. Al abordar la ocupación principal, las labores domésticas predominaban en el 58,7 % de casos y en el 54,7 % de los controles (cuadro 1).

### Factores asociados

Los análisis bivariados permitieron seleccionar las variables: contacto con fluidos, prácticas culturales tradicionales, antecedentes familiares de infección o hepatitis por virus de la hepatitis B, antecedentes sexuales y reproductivos, y vacunación contra el virus de la hepatitis B, para conformar el modelo multivariado (cuadro 2).

**Cuadro 2:** Resultados del análisis bivariado. Factores asociados con la infección por HBV en comunidades indígenas de Colombia

Variable		Casos (N = 75) n (%)	Controles (N = 150) n (%)	OR crudo (IC_95%_)	p
Contacto con fluidos					
	Cirugías previas	29 (38,7)	40 (26,7)	1,82 (1,00-3,30)	0,05
	Tatuajes occidentales	9 (12,0)	10 (6,7)	1,91 (0,73-4,97)	0,18
	Tatuajes tradicionales	6 (8,0)	16 (10,7)	0,73 (0,27-1,96)	0,53
	Extracciones dentales	12 (16,0)	28 (18,7)	0,85 (0,40-1,80)	0,67
	Transfusiones previas	9 (12,0)	15 (10,0)	1,28 (0,53-3,10)	0,59
	Comparte elementos de uso personal (cepillos, máquinas de afeitar)	8 (10,7)	18 (12,0)	0,88 (0,36-2,13)	0,77
Prácticas culturales					
	Participación en medicina tradicional	47 (62,7)	80 (53,3)	1,49 (0,83-2,68)	0,18
	Habla su lengua nativa.	51 (68,0)	87 (58,0)	1,54 (0,85-2,77)	0,15
	Participación en rituales tradicionales	32 (42,7)	53 (35,3)	1,32 (0,74-2,34)	0,34
	Recepción de alimentos masticados	9 (12,0)	12 (8,0)	1,53 (0,61-3,85)	0,37
Antecedentes de infección					
	Antecedente familiar de infección por HBV	13 (17,3)	11 (7,3)	2,65 (1,12-6,29)	0,03
	Antecedente familiar de muerte por HBV	6 (8,0)	4 (2,7)	3,18 (0,86-11,74) 0,08	
Antecedentes sexuales y reproductivos [mediana (RIC)]					
	Hijos por mujer	3 (2-5)	2 (1-4)	1,21 (1,05-1,38)	0,009
	Embarazos por mujer	4 (3-5)	3 (1-4)	1,22 (1,08-1,39)	0,002
	Número de parejas sexuales	2 (1-3)	2 (1-3)	1,02 (0,97-1,06)	0,51
	Antecedentes de vacunación				
	Vacunación contra HBV	18 (24,0)	41 (47,3)	0,83 (0,38-1,86)	0,65

OR: razón de probabilidad; HBV: hepatitis B virus

Las cirugías previas (OR = 1,82) (IC_95%_: 1,00-3,33) (p = 0,05), la presencia de tatuajes occidentales (OR = 1,91) (IC_95%_: 0,73-4,97) (p = 0,18) y otras variables, como extracciones dentales (OR = 0,85) (IC_95%_: 0,40-1,80) (p = 0,67), transfusiones previas (OR = 1,28) (IC_95%_: 0,53-3,10) (p = 0,59), compartir elementos de uso personal (OR = 0,88) (IC_95%_: 0,36-2,13) (p = 0,77) y tatuajes tradicionales (OR = 0,73) (IC_95%_: 0,27-1,96) (p = 0,53), se incluyeron en el análisis multivariado por su posible causalidad biológica en la infección por virus de la hepatitis B.

Con respecto a las variables relacionadas con prácticas culturales tradicionales, se halló una asociación (p < 0,25) entre la infección por virus de la hepatitis B y los parámetros de participación en prácticas de medicina tradicional (OR = 1,49) (IC_95%_: 0,83-2,68) (p = 0,18) y hablar la lengua nativa (OR = 1,54) (IC_95%_: 0,85-2,77) (p = 0,15). Otras variables relacionadas con las prácticas culturales, como la participación en rituales tradicionales (OR = 1,32) (IC_95%_: 0,74-2,34) (p = 0,34) y recibir alimentos previamente masticados (OR = 1,53) (IC_95%_: 0,61-3,85) (p = 0,37), no tuvieron asociación significativa, pero fueron consideradas para los modelos multivariados por su relación con posibles mecanismos de transmisión del virus.

Variables como el antecedente familiar de infección por virus de la hepatitis B (OR = 2,65) (IC_95%_: 1,12-6,29) (p = 0,03) y el antecedente familiar de muerte por virus de la hepatitis B (OR = 3,18) (IC_95%_: 0,86-11,74) (p = 0,03), cumplieron con el criterio establecido (p < 0,25) y mostraron una asociación estadísticamente significativa (p < 0,05) y, por lo tanto, ingresaron al análisis multivariado.

Con respecto a los modelos realizados solo con mujeres, las variables relacionadas con los antecedentes sexuales y reproductivos de las participantes, como el número de embarazos (OR = 1,22) (IC_95%_: 1,08-1,39) (p = 0,002) y de hijos (OR = 1,21) (IC_95%_: 1,05-1,38) (p = 0,009), mostraron una asociación estadísticamente significativa (p < 0,05) con el riesgo de infección por el virus de la hepatitis B. Aunque el número de parejas sexuales (OR = 1,02) (IC_95%_: 0,97-1,06) (p = 0,009) no cumplió con el criterio Akaike establecido (p < 0,25), se incluyó en el análisis dado su implicación potencial en la transmisión del virus.

En cuanto al antecedente de vacunación contra el virus de la hepatitis B (OR = 0,83) (IC_95%_: 0,38-1,86) (p = 0,65), se observó una mayor proporción de vacunación entre los controles comparados con los casos (p = 0,59); a pesar de esto, se incluyó en el análisis multivariado por decisión de los investigadores.

En el modelo multivariado, el antecedente familiar de hepatitis B (OR = 2,61) (IC_95%_: 1,09-6,27) continuó asociado de manera significativa con la infección por virus de la hepatitis B, es decir que tener un familiar con hepatitis B incrementó en casi 2,61 veces la posibilidad de contraer la infección (cuadro 3). Cuando la variable vacunación contra virus de la hepatitis B se incluyó en el modelo, se perdió precisión en los estimadores puntuales de las diferentes variables, debido a la disminución en el número de observaciones como resultado del gran número de datos perdidos (*missing values*). Muchas de las personas entrevistadas no sabían si habían sido vacunadas o no respondieron la pregunta.

**Cuadro 3:** Resultados del análisis multivariado (modelo 1, ajustado por cirugías previas, antecedente muerte familiar por hepatitis B, extracciones dentales, compartir elementos de uso personal y transfusiones previas). Factores asociados con la infección por HBV en comunidades indígenas de Colombia

Variable	OR crudo (IC_95%_)	p	OR ajustado (IC_95%_)	p
Antecedente familiar de hepatitis	2,65 (1,12-6,29)	0,03	2,61 (1,09-6,27)	0,03
Cirugías previas	1,82 (1,00-3,30)	0,05	1,79 (0,94-3,41)	0,07
Antecedente de muerte familiar por hepatitis	3,18 (0,86-11,74)	0,08	2,54 (0,63-10,29)	0,18
Extracciones dentales en comunidad	0,85 (0,40-1,80)	0,67	0,86 (0,40-1,81)	0,71
Compartir elementos de uso personal	0,88 (0,36-2,13)	0,77	0,92 (0,39-1,90)	0,86
Transfusiones	1,28 (0,53-3,10)	0,59	0,82 (0,31-2,19)	0,69

OR: razón de probabilidad; IC: intervalo de confianza

En las mujeres indígenas, el número de embarazos (OR = 1,61) (IC_95%_: 1,02-2,54) incrementó en 1,61 veces la posibilidad de infección por embarazo. El antecedente familiar de hepatitis B, después de ajustarlo con las diferentes variables de antecedentes reproductivos, no tuvo un efecto estadístico (OR =2,28) (IC_95%_: 0,83-6,22) (cuadro 4).

**Cuadro 4:** Resultados del análisis multivariado (modelo solo con datos de mujeres, ajustado por número de hijos, partos, nacidos vivos y antecedente familiar de hepatitis B). Factores asociados con la infección por HBV en comunidades indígenas de Colombia

Variable*	OR crudo (IC_95%_)	p	OR ajustado (IC_95%_)	p
Número de embarazos	1,22 (1,08-1,39)	0,002	1,61 (1,02-2,54)	0,04
Número de hijos	1,21 (1,05-1,38)	0,009	0,41 (0,10-2,30)	0,32
Número de partos	1,19 (1,04-1,37)	0,009	0,88 (0,42-1,81)	0,73
Número de nacidos vivos	1,21 (1,05-1,39)	0,006	1,97 (0,31-12,69)	0,47
Antecedente familiar de hepatitis	2,75 (1,06-7,12)	0,03	2,28 (0,83-6,22)	0,10

## Discusión

La infección por el virus de la hepatitis B continúa siendo un problema de salud pública alrededor del mundo ^15^, con un registro de 1,5 millones de casos nuevos por año. Los indígenas, debido a las condiciones de pobreza, discriminación e inequidad social, son poblaciones vulnerables, propensas a desarrollar diversos tipos de enfermedades y eventos en salud, como la hepatitis B ^1^^,^^9^.

La amazonía colombiana alberga un gran porcentaje de población indígena (50 % del total de la población) y presenta una de las mayores prevalencias de infección por hepatitis B ^16^. Actualmente, no hay claridad sobre los factores asociados con la transmisión horizontal del virus en adultos, porque la mayoría de los estudios previos se han llevado a cabo en niños y adolescentes ^4^^,^^7^^,^^17^.

Los resultados de esta investigación permitieron determinar una asociación estadísticamente significativa entre la presencia de antecedentes familiares de infección por virus de la hepatitis B y el riesgo de infección. Este hallazgo ha sido descrito previamente en investigaciones realizadas por Craxi *et al*., quienes determinaron la alta probabilidad de contagio cuando se convive con personas que padecen estados crónicos de la infección ^18^.

En dos estudios con poblaciones de Perú, realizados para evaluar la transmisión horizontal del virus, Cabezas *et al*. hallaron una asociación significativa entre la infección por virus de la hepatitis B y el contacto con personas dentro del núcleo familiar que padecían la enfermedad viral. Se destaca, entonces, la frecuente transmisión intrafamiliar de la hepatitis B entre convivientes portadores del virus ^19^^,^^20^.

Melo *et al*. describieron la asociación entre la infección por el virus de la hepatitis B y el antecedente de hepatitis B intrafamiliar, después de evaluar los marcadores de la enfermedad en convivientes con personas que padecían una infección crónica ^21^. Esta asociación puede connotar mecanismos de transmisión horizontal, al igual que otras variables como compartir elementos de uso personal (cepillos de dientes o máquinas de afeitar), potenciales fómite que posiblemente facilitan la transmisión del virus. Sin embargo, en este estudio no logró demostrarse su asociación con la infección ^22^^,^^23^.

El número de embarazos fue la segunda variable que tuvo una asociación estadísticamente significativa con la infección por el virus de la hepatitis B: a mayor número de embarazos de las mujeres indígenas participantes, la probabilidad de infección aumentaba en 61 % por cada embarazo. Esta variable no ha sido reportada en estudios previos realizados en población indígena. Aunque Umer *et al*. evaluaron la prevalencia de la infección por virus de la hepatitis B en mujeres del programa de control prenatal en Etiopía, no lograron demostrar una asociación con el número de embarazos ^24^. Sin embargo, esta variable puede estar relacionada con el inicio temprano de relaciones sexuales, el número de parejas y el no usar el preservativo ^25^^,^^26^.

En los casos de inicio temprano de relaciones sexuales, embarazo y posible riesgo de contagio por virus de la hepatitis B debe considerarse la probabilidad de la concomitancia de la fase de infección crónica (aumento de la expresión de HBeAg) y la gestación, lo que implica un gran riesgo de transmisión vertical ^27^^,^^28^.

En el presente estudio, se evaluaron aspectos de tipo cultural, relacionados con prácticas y rituales tradicionales (como la ingestión de alimentos previamente masticados) para determinar su posible vinculación con la transmisión e infección del virus de la hepatitis B, pero no se halló una asociación estadísticamente significativa. Sin embargo, el papel que juega este tipo de comportamientos en el riesgo de contraer la infección, se ha descrito previamente en reportes sobre poblaciones indígenas -como los escritos por Cabezas *et al*. en Perú, y Monsalve-Castillo *et al*. en Venezuela- en las cuales el intercambio de alimentos y sustancias con contenido de fluidos corporales (como la saliva) puede ser una vía de transmisión del virus, por lo que no se descarta que este mecanismo contribuya a las altas tasas de infección por el virus de la hepatitis B en indígenas ^11^^,^^12^.

Pese a que no se encontró un efecto protector, estadísticamente significativo, de la vacunación contra el virus de la hepatitis B, sí se observó un mayor porcentaje de personas vacunadas entre los controles (47,3 %) en comparación con los casos (24 %). La mediana de la edad de los participantes fue de 30 años, lo que indica que la mayoría de ellos nacieron después de 1992, año en que se inició la vacunación en Colombia. Cabe afirmar que otros estudios realizados en la amazonía colombiana y la peruana, mostraron un efecto protector de la vacunación en esta población ^8^^,^^29^^,^^30^.

Los resultados refuerzan la necesidad de abordar las desigualdades en el acceso a la vacunación, específicamente en indígenas, tal como lo destacan Ríos-Hincapié *et al*. en su abordaje de aspectos clave para la eliminación del virus de la hepatitis B, especialmente en niños ^9^.

Entre las limitaciones, se destacan la información faltante o incoherente en el SIVIGILA, lo que dificultó la localización de algunos casos. Sin embargo, algunos de estos datos pudieron corregirse con ayuda del personal de salud de cada departamento. En los casos con información incompleta, se limitó el acceso durante el periodo de estudio, por esto no se descarta la introducción de algún tipo de sesgo en la selección.

Entre los casos que tenían información de localización, se priorizó a los que vivían cerca de las capitales de los departamentos participantes, lo que también pudo haber generado algún tipo de sesgo, pero es difícil saber la dirección en la que pudieron alterarse los resultados.

La mayoría de los casos notificados e incluidos en el trabajo son de mujeres en edad fértil, diagnosticadas posiblemente mediante el programa de control prenatal, según las directrices del Ministerio de Salud y Protección Social de Colombia para la detección de enfermedades infecciosas de transmisión vertical durante el embarazo, como la hepatitis B ^31^.

Para minimizar los sesgos de información que se presentan en los estudios retrospectivos, las encuestas se aplicaron de la misma forma entre casos y controles, evitando mayor recordación en unos que en otros.

Además, no fue posible establecer el momento de infección de las personas, por lo tanto, para la mayoría de las variables, la relación temporal entre la exposición y la infección no es clara. Esto limita la posibilidad de confirmar si las asociaciones significativas encontradas son causales. Otra limitación es que algunos controles pueden corresponder a personas con infección por virus de la hepatitis B, pero con niveles bajos de antígenos (HBsAg), no detectables en la prueba rápida, o expuestas al virus pero que lograron la eliminación viral espontánea gracias a su respuesta inmunológica. Estas dos circunstancias pueden representar un sesgo y, por tanto, subestimar las asociaciones encontradas.

Entre las fortalezas del estudio, se destaca el diseño de casos y controles, de tipo observacional, analítico, con mayor validez interna que en los estudios transversales y las series de casos reportados en la literatura hasta la fecha. También, la manera en que se seleccionaron los casos y controles, pues los primeros se incluyeron a partir del registro oficial de casos reportados de hepatitis B, lo que disminuye los sesgos de selección. Los controles se incluyeron de manera concurrente con los casos, y se parearon por sexo, edad, etnia y departamento, para garantizar una mejor representación de la población de origen de los casos. Los resultados muestran una buena selección de los pares, lo que brinda confianza sobre la calidad de los controles participantes. De acuerdo con la revisión de literatura, este es el primer estudio de casos y controles pareados que solo incluye participantes indígenas de 13 etnias de diferentes regiones de Colombia, lo cual mejora la validez interna y externa del estudio.

La identificación de factores asociados con la infección, como el antecedente familiar de hepatitis B y el número de embarazos en las mujeres, corroboran lo que se ha descrito previamente sobre los mecanismos de transmisión horizontal y vertical del virus en indígenas. Como aporte adicional, si bien no era el foco de este estudio, ningún caso ni control tuvo un resultado positivo en la prueba para detección del HIV.

Finalmente, el estudio llama la atención sobre la importancia que sigue teniendo la hepatitis B en la población indígena, particularmente la que habita en la amazonía. Esto toma vigencia ante el compromiso de Colombia en la eliminación de la transmisión de la hepatitis B para el año 2030 y denota la necesidad de redoblar los esfuerzos para aumentar las coberturas de vacunación en poblaciones indígenas.
